# Factors associated with occasional and recurrent falls in Mexican community-dwelling older people

**DOI:** 10.1371/journal.pone.0192926

**Published:** 2018-02-20

**Authors:** Marcela Agudelo-Botero, Liliana Giraldo-Rodríguez, Juana Catalina Murillo-González, Dolores Mino-León, Esteban Cruz-Arenas

**Affiliations:** 1 Centro de Investigación en Políticas, Población y Salud, Facultad de Medicina, Universidad Nacional Autónoma de México, Mexico City, Mexico; 2 Departamento de Epidemiología Demográfica y Determinantes Sociales, Instituto Nacional de Geriatría, Mexico City, Mexico; 3 Subdirección de Sistemas de Información, Procesamiento y Geoestadística, Dirección General de Participación Ciudadana para la Prevención Social de la Violencia y la Delincuencia, Mexico City, Mexico; 4 Unidad de Investigación en Epidemiología Clínica, UMAE Hospital de Especialidades, Centro Médico Nacional Siglo XXI, Instituto Mexicano del Seguro Social, Mexico City, Mexico; 5 Unidad de Vigilancia Epidemiológica Hospitalaria, Investigación Sociomédica, Instituto Nacional de Rehabilitación, Mexico City, Mexico; Hospital General Dr. Manuel Gea Gonzalez, MEXICO

## Abstract

Falls are a frequent event among older adults that can cause wounds, disability, psychological disorders, and premature death. Although the large number of existing studies on the issue, few have been conducted in middle- and low-income countries. The objective of the present study is to identify the sociodemographic, medical, and functional performance factors associated with occasional and recurrent falls in Mexican older adults dwelling in community. Cross-sectional analysis of 9 598 adults ≥60 years old who participated in the fourth round (2015) of the Mexican Health and Aging Study. Bivariate tests were performed to evaluate the differences between covariates by distinct fall groups (no falls, occasional falls, and recurrent falls). Multiple logistic regressions with unadjusted and adjusted models were estimated. Approximately 46% of older adults had had at least one fall during the previous two years (one fall 16% and recurrent falls 30%). Occasional falls were only associated with being a woman; in addition to the sex, recurrent falls were strongly associated with advanced age, rural residence, bad and very bad self-perception of health status, activity-limiting pain, urinary incontinence, depression, arthritis, limitations in basic activities of daily living, and limitations in advanced activities of daily living. Falls, primarily recurrent falls, deserve to be addressed through multifactorial strategies that include different areas of intervention.

## Introduction

According to the World Health Organization, falls are defined as “inadvertently coming to rest on the ground, floor or other lower level, excluding intentional change in position to rest on furniture, wall or other objects” [1]. It has been reported that between 30% and 60% of adults ≥65 years of age who are living in the community have experienced at least one fall during the past year, and in 50% of these cases, the situation repeats itself [2,3]. Recent calculations show that falls account for 12.3 million disability-adjusted life years and are found to be among the principal causes of health burden in older adults [4].

Falls are a frequent event among older adults and can cause wounds, disabilities, psychological disorders, and premature death [1–3,5,6]. In certain cases, the consequences are permanent and irreversible. It has been shown that between 25% and 75% of older adults with a hip fracture do not recover the level of functionality that they had before the fall [7]. One-fourth of older adults restrict their daily activities due to the fear of falling again or losing their independence [7,8]. In addition to their impact on health, serious falls increase hospitalization, rehabilitation, and long-term care needs, as well as morbidity and mortality [1,7–10]. Approximately 40% of older adults with severe falls require hospitalization, and between 30% and 40% of these patients need subsequent medical attention [5].

Falls result from the complex interaction of intrinsic and extrinsic factors, including biological, behavioral, socioeconomic, and environmental factors [2,3,5,10]. Previous studies have found distinct factors associated with the risk of falling, with the most common being: being a woman, being 80 years or more, low academic level, taking four medications or more -prescribed or not-, bad self-perception of health status, having vision or hearing limitations, presenting limitations in activities of daily living (ADL), presenting limitations in instrumental activities of daily living (IADL), and suffering from some chronic diseases -like arthritis, cardiovascular diseases, chronic obstructive pulmonary disease, cancer or diabetes [2,5,11–14]. Thus, falls are closely linked to fragility, geriatric syndromes, and functional dependence, which all share some of the same basic mechanisms [8–15].

On the other hand, the factors associated with one fall (occasional falls) and multiple falls are usually different, in the sense that falling recurrently (two times or more) is associated with a greater number of conditions, primarily those related to health and functional performance [16–19]. Recurrent falls increase the risk of having more severe damage and disability [18,19], compared to those who have only fallen once [19]. Falls are a predicting factor for future falls, especially among those who fall several times [17–19]. In addition, the risk of falling increases the greater the number of risk factors exist [2,18,19]. The fall rate is 27% for older adults with zero or one risk factor, and it goes up to 78% for those with four risk factors or more [2].

Regarding international context, 80% of deaths due to falls occur in middle- and low-income countries [1,16]. However, in these countries, few studies that address the causes associated with this phenomenon [6], which is considered a public health problem [1, 16]. Specifically, in Mexico, this type of research is scarce, even though older adults (≥ 60 years) represent 10.4% of the entire population in 2015 [20] and by 2050 will comprise 21.5% of the population [21]. A portion of this population will be likely to suffer from falls or recurrent falls at any given time in their lives. For this reason, the objective of this article is to identify the sociodemographic, medical, and functional performance factors associated with occasional and recurrent falls in Mexican older adults dwelling in community.

## Methods

### Setting and participants

This is a cross-sectional study that analyzed the data from the fourth round (2015) of the Mexican Health and Aging Study (MHAS) [22]. The MHAS is the representative of the population ≥50 years at a national level, and its general objective was to examine the aging process, diseases, and the burden of disability. The data is free access and it is available at the following URL: http://www.mhasweb.org/

For this study, the sample consisted of 9 598 adults ≥60 years who directly responded to the question: “Have you fallen in the las two years?” whose answer was dichotomous (yes or no). The people who answered affirmatively were later asked, how many times has this happened more or less? Thereby, three groups were created: with no falls, with one fall, and with recurring falls (≥2 falls).

### Covariates

The co-variables were divided into three large groups: sociodemographic, medical, and functional.

The sociodemographic variables included: sex (male, female); age (60–79, ≥80); education (<10, ≥10 years); marital status (married/living-in, unmarried/single); employment (employed, unemployed) and place of residence (urban, rural).

The medical variables were: self-perception of health (very good or good, regular, very poor or poor). Permanent activity-limiting pain (questions: Does this pain limit your usual activities such as household chores or your job? responses were categorized as yes or no). Urinary incontinence (questions: During the last two years, have you frequently had any of the following problems or inconveniences: incontinence when coughing, sneezing, picking something up, or exercising; or incontinence when you had the urge to urinate but couldn’t reach the bathroom in time? responses were categorized as yes or no). Vision problems (question: How is your vision (with glasses)? responses were categorized as yes or no). Hearing problems (question: How is your hearing ability (with a hearing aid)? responses were categorized as yes or no). Cognitive decline (typical values on the Transcultural Cognitive Test [23], which was based on the following eight domains: orientation, attention, verbal learning, evoking memory, spatial ability, visual memory, executive function, and numeracy were used; answers were classified as: no deterioration, light deterioration, severe deterioration, or inconclusive test. Depression (measured using a modified version of the United States Center for Epidemiological Studies—Depression Scale (CES-D), which has been validated for the MHAS population [24]. This scale is based on symptoms of geriatric depression, with affirmative response to at least five of the nine questions being considered positive for depression; responses were categorized as yes or no). Self-report of principal chronic health conditions (hypertension, diabetes, cancer, chronic obstructive pulmonary disease, heart attack, stroke, angina and arthritis; responses were categorized as yes or no).

The following were the functional variables: Limitations in ADL (difficulty in at least one of the following activities: dressing, walking, bathing, eating, going to bed, or using the toilet; responses were categorized as yes or no). Limitations in IADL (difficulty in at least one of the following: preparing food, shopping, taking medications, or managing money; responses were categorized as yes or no). Advanced activities of daily living (AADL) (difficulty in at least one of the following: walking one block; climbing one floor of stairs without resting; lifting or carrying objects that weigh more than 5 kg; pulling or pushing large objects; picking up a coin from the table; postural behaviors inclusive of inclining, getting down on one’s knees, bending or squatting down; or extending one’s arms higher than one’s shoulders; responses were categorized as yes or no).

### Statistical analysis

The variables are described using means (standard deviation [SD]) for continuous variables and frequencies and percentages for categorical variables. Bivariate tests were performed to evaluate the differences between covariates by distinct fall groups (no falls, occasional falls and recurrent falls). Multiple logistic regressions with unadjusted and adjusted models (for sociodemographic, medical and functional covariables) were performed, and odds ratios (OR) with 95% confidence intervals (CI) were obtained. Data were analyzed using the Stata version 13.1^®^ program (Stata Corp, College Station, Texas, USA).

### Ethical issues

The MHAS study protocol and instruments were approved by the Institutional Review Board or Ethics Committee of the University of Texas Medical Branch, the National Institute of Statistics and Geography in Mexico, and the National Institute of Public Health in Mexico. Oral informed consent was requested in accordance with the ethical principles for research on humans in the Declaration of Helsinky [22]. In addition to the analysis of this study approval by the Ethics Committee and Research of the National Institute of Geriatrics of Mexico was obtained (DI-PI-002/15).

## Results

**Fig 1:**
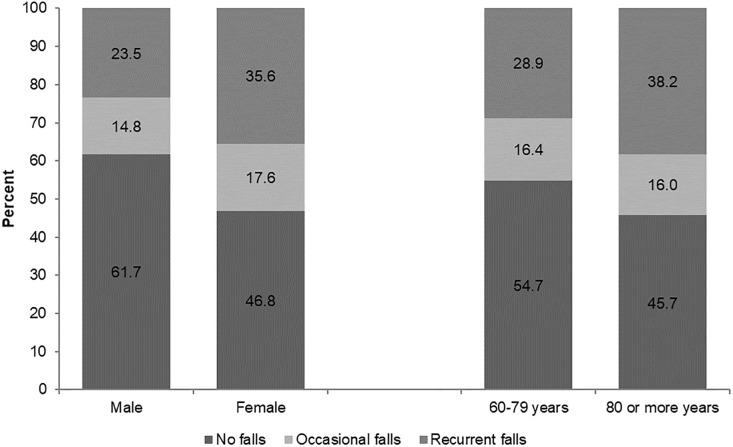
Self-reported incidence of falls over the last two years in older Mexican adults by sex and age group. Mexico, 2015.

Out of the 9 598 people surveyed, 55.5% were female, and 86% were between 60 and 79 years of age. Approximately 46.5% had at least one fall during the previous two years (occasional falls 16.4% and recurrent falls 30.2%). The average number of falls was 2.9 (SD = 3.8), with females experiencing a greater number of falls (3.3; SD = 4) than males (2.8; SD = 3.8). The numbers of falls were similar across age groups (60–69 years: 2.9; SD = 3.9; ≥80 years: 3.2; SD = 3.3). The greatest percentage of total falls (occasional and recurrent) occurred in women and in the most advanced age group (Fig 1). The average age of all those who experienced a fall was 71.4 (SD = 7.7) (occasional falls: 70.7; SD = 7.6; recurrent falls: 71.7; SD = 7.7). In 36.5% of cases, medical treatment was necessary due to the wounds suffered.

The different characteristics of the older adult population by falling experience are shown in Table 1 (no falls, occasional falls, and recurrent falls). The population under study was generally concentrated between 60–79 years, with low academic level, living in free union or married, and most of them from the urban area. The feminine sex predominated in the occasional and recurrent falls group. When comparing the health conditions and functional performance of all three fall groups, it was observed that older adults with recurrent falls got the worst percentages, followed by adults with occasional falls and no falls. On the other hand, it was observed in the bivariate analysis that there were statistically significant differences between the groups in almost all the covariables, except cancer, heart attack, and stroke.

**Table 1:** Descriptive analysis of the sample. Mexico, 2015.

Characteristics	No falls		Occasional falls		Recurrent falls		*χ*2 *p* value
	(n = 5 132)		(n = 1 570)		(n = 2 896)		
	N	(%)	n	(%)	n	(%)	
***Sociodemographic variables***							
Sex							
Male	2637	(51.4)	632	(40.3)	1002	(34.6)	<0.001
Female	2495	(48.6)	938	(59.7)	1894	(65.4)	
Age group							
60–79	4516	(88.0)	1354	(86.2)	2381	(82.2)	<0.001
> = 80	616	(12.0)	216	(13.8)	515	(17.8)	
Education							
<10 years	4406	(87.0)	1394	(89.5)	2641	(91.9)	<0.001
> = 10 years	657	(13.0)	164	(10.5)	234	(8.1)	
Marital status							
Married/living-in	3387	(66.0)	986	(62.8)	1666	(57.0)	<0.001
Unmarried/single	1745	(34.0)	584	(37.2)	1230	(42.5)	
Employment							
Employed	1785	(35.1)	484	(31.0)	721	(25.1)	<0.001
Unemployed	3299	(64.9)	1079	(69.0)	2152	(74.9)	
Place of residence							
Rural	913	(17.8)	252	(16.1)	627	(21.7)	<0.001
Urban	4219	(82.2)	1318	(83.9)	2269	(78.3)	
***Medical variables***							
Self-perception of health status							
Very good or good	1781	(34.7)	464	(29.6)	631	(21.8)	<0.001
Regular	2715	(52.9)	870	(55.4)	1570	(51.2)	
Very poor or poor	634	(12.4)	235	(15.0)	695	(24.0)	
Permanent activity-limiting pain							
Yes	750	(14.6)	265	(16.9)	848	(29.3)	<0.001
No	4381	(85.4)	1305	(83.1)	2047	(70.7)	
Urinary incontinence							
Yes	1330	(25.9)	475	(30.3)	1216	(42.0)	<0.001
No	3798	(74.1)	1095	(69.7)	1679	(58.0)	
Vision problems							
Yes	2346	(45.8)	747	(47.6)	1577	(54.5)	<0.001
No	2781	(54.2)	821	(52.4)	1317	(45.5)	
Hearing problems							
Yes	1786	(34.9)	585	(37.4)	1264	(43.8)	<0.001
No	3326	(65.1)	980	(62.6)	1625	(56.2)	
Cognitive decline							
No deterioration	4145	(83.6)	1252	(82.2)	2212	(78.4)	<0.001
Light deterioration	216	(4.4)	90	(5.9)	149	(5.3)	
Severe deterioration	14	(0.3)	2	(0.1)	7	(0.2)	
Inconclusive test	584	(11.8)	180	(11.8)	455	(16.1)	
Depression							
Yes	1322	(26.0)	471	(30.3)	1287	(44.9)	<0.001
No	3760	(74.0)	1085	(69.7)	1580	(55.1)	
Hypertension							
Yes	2522	(49.2)	836	(53.4)	1663	(57.5)	<0.001
No	2606	(50.8)	731	(46.6)	1229	(42.5)	
Diabetes							
Yes	1243	(24.3)	409	(26.1)	873	(30.2)	<0.001
No	3882	(75.7)	1159	(73.9)	2020	(69.8)	
Cancer							
Yes	117	(2.3)	47	(3.0)	87	(3.0)	0.088
No	5009	(97.7)	1523	(97.0)	2805	(97.0)	
Chronic obstructive pulmonary							
Yes	278	(5.4)	111	(7.1)	232	(8.0)	<0.001
No	4850	(94.6)	1459	(92.9)	2664	(92.0)	
Heart attack							
Yes	206	(4.0)	72	(4.6)	140	(4.8)	0.200
No	4923	(96.0)	1497	(95.4)	2755	(95.2)	
Angina							
Yes	343	(6.7)	120	(7.7)	288	(10.0)	<0.001
No	4769	(93.3)	1448	(92.3)	2602	(90.0)	
Stroke							
Yes	99	(1.9)	28	(1.8)	84	(2.9)	0.008
No	5025	(98.1)	1540	(98.2)	2812	(97.1)	
Rheumatoid arthritis							
Yes	744	(14.5)	237	(15.1)	692	(23.9)	<0.001
No	4379	(85.5)	1332	(84.9)	2199	(76.1)	
***Functional variables***							
Limitations in ADL							
Yes	863	(26.5)	335	(29.7)	941	(39.7)	<0.001
No	2396	(73.5)	793	(70.3)	1431	(60.3)	
Limitations in IADL							
Yes	828	(16.2)	296	(18.9)	762	(26.4)	<0.001
No	4284	(83.8)	1273	(81.1)	2126	(73.6)	
Limitations in AADL							
Yes	2821	(55.2)	1011	(64.4)	2184	(75.6)	<0.001
No	2291	(44.8)	558	(35.6)	704	(24.4)	

ADL, Activities of Daily Living; IADL, Instrumental Activities of Daily Living; AADL, Advanced activities of daily living.

Sociodemographic, medical, and functional variables associated with occasional and recurrent falls are shown in Table 2. Once the models for all covariables were adjusted, it was found that the sole fact of being a woman was significantly associated with occasional falls (OR, 1.57; 95% CI, 1.37–1.59; *p* = <0.001).

**Table 2:** Multiple logistic regression with unadjusted and adjusted models for sociodemographic, medical and functional variables associated with occasional falls and recurrent falls.

Explanatory variables (reference)	Crude odds ratio (95%CI)				Adjusted odds ratio (95% CI)			
	Occasional falls		Recurrent falls		Occasional falls		Recurrent falls	
	OR	IC 95%	OR	IC 95%	OR	IC 95%	OR	IC 95%
***Sociodemographic variables***								
Sex: Female (Male)	1.57*	(1.40–1.76)	2.00*	(1.82–2.20)	1.57*	(1.37–1.79)	1.68*	(1.50–1.88)
Age group: 80+ (60–79)	1.17	(0.99–1.38)	1.59*	(1.40–1.80)	1.17	(0.98–1.40)	1.35*	(1.17–1.56)
Education: <10 year (≥10 years)	1.27**	(1.06–1.52)	1.68*	(1.44–1.97)	1.10	(0.91–1.34)	1.03	(0.87–1.23)
Marital status: Unmarried/single (Married/living-in)	1.15**	(1.02–1.29)	1.43*	(1.31–1.57)	0.97	(0.85–1.10)	1.10	(0.99–1.22)
Employment: Unemployed (Employed)	1.21**	(1.07–1.36)	1.62*	(1.46–1.79)	0.97	(0.84–1.11)	1.10	(0.98–1.23)
Place of residence: Rural (Urban)	0.88	(0.76–1.03)	1.28*	(1.14–1.43)	0.88	(0.75–1.03)	1.30*	(1.15–1.47)
***Medical variables***								
Self-perception of health status: Very poor or poor (Very good or good)	1.42*	(1.19–1.71)	3.09*	(2.69–3.56)	1.22	(0.99–1.51)	1.64*	(1.39–1.94)
Self-perception of health status: Regular (Very good or good)	1.23**	(1.08–1.40)	1.63*	(1.46–1.82)	1.13	(0.98–1.29)	1.22**	(1.08–1.38)
Permanent activity-limiting pain: Yes (No)	1.19**	(1.02–1.38)	2.42*	(2.17–2.71)	0.92	(0.77–1.10)	1.34*	(1.17–1.53)
Urinary incontinence: Yes (No)	1.24**	(1.09–1.40)	2.07*	(1.88–2.28)	1.12	(0.98–1.28)	1.52*	(1.37–1.69)
Vision problems: Yes (No)	1.08	(0.96–1.21)	1.42*	(1.30–1.56)	1.01	(0.89–1.14)	1.11	(1.00–1.23)
Hearing problems: Yes (No)	1.11	(0.99–1.25)	1.45*	(1.32–1.59)	1.09	(0.96–1.23)	1.19**	(1.07–1.32)
Cognitive decline: Inconclusive test (No deterioration)	1.02	(0.85–1.22)	1.46*	(1.28–1.67)				
Cognitive decline: Light test (No deterioration)	1.38**	(0.17–1.78)	1.29**	(1.04–1.60)				
Cognitive decline: Severe (No deterioration)	0.47	(0.11–2.08)	0.94	(0.38–2.33)				
Depression: Yes (No)	1.24**	(1.09–1.40)	2.32*	(2.10–2.55)	1.04	(0.91–1.19)	1.53*	(1.37–1.70)
Hypertension: Yes (No)	1.18**	(1.06–1.32)	1.40*	(1.28–1.53)	1.06	(0.94–1.20)	1.06	(0.96–1.18)
Diabetes: Yes (No)	1.10	(0.97–1.26)	1.35*	(1.22–1.50)	1.02	(0.89–1.17)	1.13**	(1.01–1.27)
Cancer: Yes (No)	1.32	(0.94–1.86)	1.33	(1.00–1.76)				
Chronic obstructive pulmonary disease: Yes (No)	1.33**	(1.06–1.67)	1.52*	(1.27–1.82)	1.22	(0.96–1.54)	1.13	(0.93–1.37)
Heart attack: Yes (No)	1.15	(0.87–1.51)	1.21	(0.98–1.51)	1.05	(0.78–1.43)	0.97	(0.76–1.24)
Angina: Yes (No)	1.15	(0.93–1.43)	1.54*	(1.31–1.81)	1.06	(0.84–1.34)	1.16	(0.96–1.40)
Stroke: Yes (No)	0.92	(0.60–1.41)	1.52**	(1.13–2.04)	0.85	(0.55–1.30)	1.18	(0.86–1.62)
Rheumatoid arthritis: Yes (No)	1.05	(0.89–1.23)	1.85*	(1.65–2.08)	0.87	(0.74–1.03)	1.26*	(1.11–1.43)
***Functional variables***								
Limitations in ADL: Yes (No)	1.17**	(1.01–1.36)	1.83*	(1.63–2.05)	1.12	(0.95–1.32)	1.61*	(1.42–1.83)
Limitations in IADL: Yes (No)	1.20**	(1.04–1.39)	1.85*	(1.66–2.07)	1.01	(0.84–1.21)	1.09	(0.95–1.25)
Limitations in AADL: Yes (No)	1.47*	(1.31–1.65)	2.52*	(2.28–2.79)	1.24	(0.99–1.54)	1.47*	(1.22–1.78)

p-value:

* <0.001,

** <0.05

ADL, Activities of Daily Living; IADL, Instrumental Activities of Daily Living; AADL, Advanced activities of daily living.

In the case of older adults with recurrent falls, practically all covariables was significantly associated in the crude model, however, the number of associated factors decreased as it was adjusted. As for sociodemographic variables, sex, age, and place of residence were associated with recurrent falls. The health variables that were significantly associated with recurrent falls were: self-perception of health status (the OR being greater for those who had a bad or very bad health perception), permanent activity-limiting pain, urinary incontinence, vision problems, hearing problems, depression, also diseases such as diabetes and arthritis. As for functionality variables, it was observed that presenting ADL and AADL was strongly associated with recurrent falls, as well.

## Discussion

The fall prevalence in the analyzed sample was 46.5%; 16.4% reported to have had occasional falls and 30.2% recurrent falls. These results are consistent with what was previously described in literature, despite there being differences in the study design, analyzed populations, and accounted periods of exposition [18,25,26], which does not allow for direct comparisons. In this case, there was an inquiry at MHAS about the fall history for the two previous years, which would explain the produced prevalence being slightly higher than in other researches.

Even though the risk factors related to occasional and recurrent falls have been previously analyzed [3, 17–19, 27], this is the first study in Mexico with representativeness at a national level that takes this criteria into account in order to assess the sociodemographic, medical, and functional variables associated with falls in the older adult population dwelling in community. Moreover, the analysis was divided into occasional (one time) and recurrent (two times or more) falls, which allows to distinguish the different effects of the covariables, thereby contributing evidence that matches the characteristics of the study population, which will serve as a basis for the design and adequacy of prevention and attention interventions.

A first finding, which is in line with what was reported in other studies, is that a lesser number of factors were associated with occasional falls, while recurrent falls had a relation with a greater number of variables [3,18,19,27]. It has been raised, thereon, that occasional falls respond to extrinsic events of a fortuitous nature and isolated accidents, while older adults with recurrent falls show a multifactorial and complex risk profile that increases the probability of falling several times, and whose causes are mostly intrinsic [2,3,18,27].

After adjusting the regression models, only one variable (sex) was significantly associated with occasional falls (and with recurrent falls, as well), a result that has been widely discussed in other investigations. In this respect, evidence points towards women standing a greater risk of falling due to the social, economic, and accumulated health disadvantages throughout the course of life, as well as more activity in and outside the house, compared to men [2,3, 14, 18,19,27].

In contrast, sociodemographic, medical, and functional variables showed a strong association with recurrent falls, which matches what is reported in literature [3, 8,18,19,27]. In addition to being a woman, recurrent falls were associated with advanced age and with rural residence. Being 80 years of age or more is an important recurrent fall predictor due to the fact that it is in this age group where there is a greater prevalence of functional limitations, as well as the confluence of different diseases that affect balance and muscular strength, making adults more prone to having several falls [3,8,18,19]. Little is known in regard to recurring falls in older adults from rural areas, although it is possible this is linked to worse health and mobility performance [28].

On the other hand, the medical variables that exhibited more statistically significant associations with recurrent falls were: bad and very bad self-perception of health status, permanent activity-limiting pain, urinary incontinence, depression, and arthritis. In all these factors, considered to be geriatric syndromes, there are common underlying elements closely related to recurrent falls. In this way, for example, it has been found that a bad and very bad self-perception of health status, as well as depression, lead to loss of autonomy and an increase in feelings of insecurity, anxiety, isolation and fear of falling again [18,19,29]. In this study, 24% of adults with recurrent falls had a bad or very bad self-perception of health status, and around 45% suffered from depression, higher values than the ones observed in both comparison groups (without falls and with occasional falls).

Likewise, it was revealed that the permanent activity-limiting pain was associated with recurrent falls, an aspect that gains greater importance if we take into consideration that the only chronic illness that also showed a statistically significant association was arthritis. Older adults with arthritis often suffer walking disorders, fatigue, rigidness in the lower extremities, and neuromuscular problems, accompanied by constant, strong pains that interfere with cognition and executive function [18, 30,31].

Regarding urinary incontinence, there is no evidence on its link to recurrent falls, although it is known that older adults who present this geriatric syndrome increase the risk of falling, and it is more common in people with physical limitations; it is also known that a greater number of affected functional levels contributes to an increase in falls or urinary incontinence [32]. In this sense, it is recommended that future investigations explore this aspect in more depth, which allows to distinguish the role urinary incontinence plays in falls, especially recurrent falls, more clearly.

Finally, the group of described health conditions directly impacts the functional development of older adults, which in turn has an influence in recurrent falls. Some studies show that health deterioration happens almost simultaneously with the loss of functional capacities [8,18,19,27], which results in an increase in dependency and an increase in falling repeatedly [2]. Researches have also revealed a bidirectional relation between recurrent falls and physical dependence, that this association increases when the falls are more frequent and more severe [2,18], or rather when there is a greater number of basic, instrumental or advanced limitations in everyday life.

### Limitations

Because one of the objectives of the MHAS was to understand the burden of disability, this survey was used to collect information on a wide range of variables that are associated with falls [2,3,5]. In addition, a sampling scheme that allowed for national representativeness was employed, if it gave robust enough results [22]. Still, this study has certain limitations. One of these relates to the effect of time interval on memory of a fall, with findings discordant in this respect [33–37]. For example, it has been reported that persons have more trouble remembering a fall after 3 or 6 months in comparison with remembering one that occurred 12 months prior [36,37]. In another study, there appeared to be no difference in the likelihood of remembering falls after 3 months versus after 12 months [37]. As a result of this, a limitation of this study is possible memory bias, which is incurred upon asking about the number of falls experienced in the two years prior to the study, although there is also evidence that might suggest that these findings are not as important as those shown in a study in which the proportion of people who reported having falls in two consecutive years was similar enough to be addressed as well when using a retrospective methodology as when using a prospective one [34]. Even though these results are cross-sectional, they are nevertheless useful for developing hypotheses and future research ideas to longitudinally explore health problems in this population.

## Conclusion

Falls, primarily recurrent falls, deserve to be addressed through multifactorial strategies that include several fields: sociodemographic, clinical, and functional. It is suggested to move on to the evaluation of demographic tools that enable to measure the risk of falls in the Mexican older adult population, taking the wide heterogeneity of this age group into account, as well as the socioeconomic and cultural context that characterizes the country.

## References

[1] World Health Organization. Global Report on Falls Prevention in Older Age. Geneva: Switzerland, 2007.

[2] Dionyssiotis Y. Analyzing the problem of falls among older people. Int J Gen Med. 2012; 5:805–813. doi: 10.2147/IJGM.S32651 23055770 10.2147/IJGM.S32651 PMC3468115

[3] Stalenhoef PA, Diederiks JP, Knottnerus JA, Kester AD, Crebolder HF. A risk model for the prediction of recurrent falls in community-dwelling elderly: a prospective cohort study. J Clin Epidemiol. 2002; 55:1088–1094. 12507672 10.1016/s0895-4356(02)00502-4

[4] Prince MJ, Wu F, Guo Y, Gutierrez Robledo LM, O’Donnell M, Sullivan R, et al The burden of disease in older people and implications for health policy and practice. Lancet. 2015; 385:549–562. doi: 10.1016/S0140-6736(14)61347-7 25468153 10.1016/S0140-6736(14)61347-7

[5] Tromp AM, Pluijm SM, Smit JH, Deeg DJ, Bouter LM, Lips P. Fall-risk screening test: a prospective study on predictors for falls in community-dwelling elderly. J Clin Epidemiol. 2001; 54:837–844. 11470394 10.1016/s0895-4356(01)00349-3

[6] Kalula SZ, Ferreira M, Swingler GH, Badri M. Risk factors for falls in older adults in a South African Urban Community. BMC Geriatr. 2016; 16:51 doi: 10.1186/s12877-016-0212-7 26912129 10.1186/s12877-016-0212-7 PMC4766747

[7] McClure RJ, Hughes K, Ren C, McKenzie K, Dietrich U, Vardon P, et al The population approach to falls injury prevention in older people: findings of a two community trial. BMC Public Health. 2010; 10:79 doi: 10.1186/1471-2458-10-79 20167124 10.1186/1471-2458-10-79 PMC2836986

[8] Manrique-Espinoza, Salinas-Rodríguez A, Moreno-Tamayo K, Téllez-Rojo MM. Functional dependency and falls in elderly living in poverty in Mexico. Salud Pública Méx. 2011; 53:26–33 (in Spanish). 21340137

[9] Gannon B, O’Shea E, Hudson E. Economic consequences of falls and fractures among older people. Ir Med J. 2008; 101:170–173. 18700509

[10] Stevens JA, Corso PS, Finkelstein EA, Miller TR. The costs of fatal and non-fatal falls among older adults. Inj Prev. 2006; 12:290–295. doi: 10.1136/ip.2005.011015 17018668 10.1136/ip.2005.011015 PMC2563445

[11] Feldman F, Chaudhury H. Falls and the physical environment: a review and a new multifactorial falls-risk conceptual framework. Can J Occup Ther. 2008; 75:82–95. doi: 10.1177/000841740807500204 18510252 10.1177/000841740807500204

[12] King M. Falls In: Halter JB, Ouslander JG, Tinetti ME, Studenski S, High KP, Asthana A, eds. Hazzard’s Geriatric Medicine and Gerontology. 6th edn New York: McGraw-Hill Companies, Inc.; 2009 p. 659–69.

[13] Nachreiner NM, Findorff MJ, Wyman JF, McCarthy TC. Circumstances and consequences of falls in community-dwelling older women. J Womens Health. 2007; 16:1437–1446. 10.1089/jwh.2006.0245 18062759

[14] Deandrea S, Lucenteforte E, Bravi F, Foschi R, La Vecchia C, Negri E. Risk factors for falls in community-dwelling older people: a systematic review and meta-analysis. Epidemiology. 2010; 21:658–668. doi: 10.1097/EDE.0b013e3181e89905 20585256 10.1097/EDE.0b013e3181e89905

[15] Samper-Ternent R, Karmarkar A, Graham J, Reistetter T, Ottenbacher K. Frailty as a predictor of falls in older Mexican Americans. J Aging Health. 2012; 24:641–653. doi: 10.1177/0898264311428490 22187090 10.1177/0898264311428490 PMC3448373

[16] World Health Organization. Falls. Fact sheet N°344; 2007 [cited 2016 Jul 13]. http://www.who.int/mediacentre/factsheets/fs344/en/

[17] Hestekin H, O’Driscoll T, Stewart J, Kowal P, Peltzer K, Chatterji S. Measuring prevalence and risk factors for fall-related injury in older adults in low- and middle-income countries: results from the WHO Study on Global AGEing and Adult Health (SAGE) [monograph on the Internet]. Geneva: World Health Organization; 2013. [Cited 14 Jul 2016]. http://www.who.int/healthinfo/sage/SAGEWorkingPaper6_Wave1Falls.pdf

[18] Abreu DR, Azevedo RC, Silva AM, Reiners AA, Abreu HC. Factors associated with recurrent falls in a cohort of older adults. Cien Saude Colet. 2016; 21:3439–3446. doi: 10.1590/1413-812320152111.21512015 27828577 10.1590/1413-812320152111.21512015

[19] Gassmann KG, Rupprecht R, Freiberger E, IZG Study Group. Predictors for occasional and recurrent falls in community-dwelling older people. Z Gerontol Geriatr. 2009; 42:3–10. doi: 10.1007/s00391-008-0506-2 18327690 10.1007/s00391-008-0506-2

[20] National Institute of Statistics and Geography. Intercensal Survey 2015; 2016 [cited 2016 Jul 08]. http://www.inegi.org.mx/est/contenidos/Proyectos/encuestas/hogares/especiales/ei2015/ (in Spanish).

[21] National Population Council. Population projection. Mexico 2010–2050; 2016 [cited 2016 Jul 08]. http://www.conapo.gob.mx/es/CONAPO/Proyecciones_Datos (in Spanish).

[22] University of Texas Medical Branch (UTMB)/University of Wisconsin/ National Institute of Statistics and Geography (INEGI)/ National Institute of Geriatrics (INGER)/ National Institute of Public Health (INSP). Mexican Health and Aging Study (MHAS); 2016 [cited 2016 Jul 11]. http://www.mhasweb.org/DataDocumentationNew.aspx

[23] Mejía-Arango S, Wong R, Michaels-Obregón A. Normative and standardized data for cognitive measures in the Mexican Health and Aging Study. Salud Publica Méx. 2015; 57:90–96. 10.21149/spm.v57s1.7594 PMC4698135 26172239

[24] Aguilar-Navarro SG, Fuentes-Cantú A, Ávila-Funes JA, García-Mayo EJ. Validity and reliability of the screening questionnaire for geriatric depression used in the Mexican Health and Age Study. Salud Pública Méx. 2007; 49:256–262 (in Spanish). 17710274 10.1590/s0036-36342007000400005

[25] Aranda MP, López-Ortega M, Gutiérrez-Robledo LM. Prevalence and determinants of falls among older Mexicans: Findings from the Mexican National Health and Nutrition Survey In: Vega WA, Markides K, Ángel JL et al, eds. Challenges of Latino Aging in the Americas, Gewerbestrasse: Springer; 2015 p. 171–88.

[26] Reyes-Ortiz CA, Al Snih S, Markides KS. Falls among elderly persons in Latin America and the Caribbean and among elderly Mexican-Americans. Rev Panam Salud Publica. 2005; 17:362–369. 16053646 10.1590/s1020-49892005000500008

[27] Curcio CL, Gómez F, Osorio JL, Rosso V. Recurrent falls in the elderly. Acta Med Colomb. 2009; 34:103–110 (in Spanish).

[28] Manrique-Espinoza B, Salinas-Rodríguez A, Salgado de Snyder N, Moreno-Tamayo K, Gutiérrez-Robledo LM, Avila-Funes JA. Frailty and Social Vulnerability in Mexican Deprived and Rural Settings. J Aging Health. 2016; 28:740–752. doi: 10.1177/0898264315609909 26464372 10.1177/0898264315609909

[29] Launay C, De Decker L, Annweiler C, Kabeshova A, Fantino B, Beauchet O. Association of depressive symptoms with recurrent falls: a cross-sectional elderly population based study and a systematic review. J Nutr Health Aging. 2013; 17:152–157. doi: 10.1007/s12603-012-0370-z 23364494 10.1007/s12603-012-0370-z PMC12879618

[30] Stubbs B, Binnekade T, Eggermont L, Sepehry AA, Patchay S, Schofield P. Pain and the Risk for Falls in Community-Dwelling Older Adults: Systematic Review and Meta-Analysis. Arch Phys Med Rehabil. 2014; 95: 175–187. doi: 10.1016/j.apmr.2013.08.241 24036161 10.1016/j.apmr.2013.08.241

[31] Leveille SG, Jones RN, Kiely DK et al Chronic musculoskeletal pain and the occurrence of falls in an older population. JAMA. 2009; 302: 2214–2221. doi: 10.1001/jama.2009.1738 19934422 10.1001/jama.2009.1738 PMC2927855

[32] Loharuka S, Barrett J, Roe B. Incontinence and falls in older people: is there a link? Nurs Times. 2005; 101:52–54. 16329277

[33] Ganz DA, Higashi T, Rubenstein LZ. Monitoring falls in cohort studies of community-dwelling older people: effect of the recall interval. J Am Geriatr Soc. 2005; 53:2190–2194. doi: 10.1111/j.1532-5415.2005.00509.x 16398908 10.1111/j.1532-5415.2005.00509.x

[34] Fleming J, Matthews FE, Brayne C. Falls in advanced old age: recalled falls and prospective follow-up of over-90-year-olds in the Cambridge City over-75s Cohort study. BMC Geriatrics. 2005; 8:6. 10.1186/1471-2318-8-6 PMC2292187 18366645

[35] Hale WA, Delaney MJ, Cable T. Accuracy of patient recall and chart documentation of falls. J Am Board Fam Pract. 1993; 6:239–242. 8503294

[36] Cummings SR, Nevitt MC, Kidd S. Forgetting falls: The limited accuracy of recall of falls in the elderly. J Am Geriatr Soc. 1988; 36:613–616. 3385114 10.1111/j.1532-5415.1988.tb06155.x

[37] Haga H, Yasumura S, Niino N, Ueno H, Oshima M, Higuchi Y. An examination of two reporting methods of falls among the elderly living in the community. Nippon Koshu Eisei Zasshi. 1996; 43:983–988. 9033213

